# Intraoperative Nodule Localization in Non-Small-Cell Lung Cancer: Existing and Emerging Techniques

**DOI:** 10.3390/cancers18121915

**Published:** 2026-06-12

**Authors:** Aidan Aicher, Jerica Tidwell, Sunil Singhal, Jarrod Predina

**Affiliations:** Division of Thoracic Surgery, Department of Surgery, Perelman School of Medicine at the University of Pennsylvania, Philadelphia, PA 19104, USA; aidan.aicher@pennmedicine.upenn.edu (A.A.);

**Keywords:** lung cancer, nodule localization, intraoperative molecular imaging

## Abstract

As thoracic surgeons more frequently address smaller lung lesions and perform lung-sparing resections, their objective is to resect an adequate specimen and margin without removing excess healthy lung tissue. Although perioperative lung nodule localization has been in practice for decades, the existing and emerging techniques used for the identification of targeted and occult lesions are more widely utilized today than they were in the past. In this review, we detail the logic behind this increase in use, classify the techniques into preoperative and intraoperative categories, and define the specific modalities available. Where applicable, we review the published data comparing techniques, detailing efficacy and safety. In the preoperative space, we describe standard computed tomography (CT)-guided localization, virtual-assisted lung mapping, electromagnetic navigation bronchoscopy, robotic-assisted bronchoscopy, and novel fiducial markers. In the intraoperative space, we describe classical localization techniques, novel applications of intraoperative cone-beam CT, and fluorescence-guided surgery and intraoperative molecular imaging (IMI). Lastly, we review emerging approaches for intraoperative molecular imaging including a report on agents in early-stage clinical trials and a brief survey of promising preclinical models. With each approach mentioned, we analyze the potential benefits and hazards, and appraise the evidence for (or against) the use of any specific modality.

## Simple Summary

Advances in lung cancer screening have led to the increased diagnosis of smaller, more difficult to detect lung lesions. At the same time, minimally invasive and robotic thoracic surgery has entered the mainstream. While these approaches have advantages, the “gold standard” of the direct palpation of suspected lung lesions is more difficult. As these trends emerge, approaches for preoperative and intraoperative nodule localization including preoperative marking and intraoperative molecular imaging are more frequently utilized. In this review, we cover the range of options available to clinicians who seek to localize pulmonary nodules, whether in the preoperative/interventional space or intraoperatively in the context of surgical resection.

## 1. Introduction

Despite advances in screening, diagnosis, and treatment, lung cancer remains the leading cause of cancer death in the US, surpassing over 120,000 per year. A total of 87% of all cases of lung cancer are non-small-cell lung cancer (NSCLC). The mainstay of treatment of early-stage NSCLC is surgical resection. With the advent of modern low-dose CT (LDCT) screening, early-stage diagnoses are increasing: as of 2024, approximately 42% of yearly NSCLC diagnoses are local, an increase of over 10% over the course of five years [1,2]. Additionally, phase III clinical trials including JCOG0802 and CALGB 140503 assessing the safety and efficacy of sublobar resection versus lobectomy in peripheral NSCLC ≤ 2 cm provide evidence that suggests limited resection may be appropriate in well-selected patients [3,4]. In this landscape, surgeons are more frequently operating on smaller nodules and considering more selective and technically challenging resections. Even further, the increased utilization of minimally invasive and robotic-assisted resection limits the ability of surgeons to intraoperatively palpate the target lesion or other suspicious parenchyma, taking away perhaps the most reliable nodule identification technique available for surgeons.

Given the state of lung cancer surgery in 2026, the localization of target nodules is critical. Localization plays important roles in intraoperative determination of extent of resection, adequate nodal dissection, and assessment of the margin needed to achieve R_0_ resection. In addition to localizing a diagnosed lesion, intraoperative lesion detection in the surgical resection of cancer is crucial to ensure synchronous lesions are not neglected. This imperative is compounded in cases of pulmonary metastasectomy for a distant primary cancer. In studies focused on metastasectomy, published rates of ipsilateral nodules “missed” on preoperative imaging approach 20% and are more often detected upon open resection [5,6]. In the past 10 years, advances in preoperative and intraoperative localization techniques have improved the surgeon’s ability to assess target lesions and detect previously undiagnosed regions of concern.

The objective of this descriptive review is to cover the tools at a thoracic surgeon’s disposal to localize lesions. To categorize the available options in a structured fashion, localization methods are structured based on their relation to the operation: preoperative versus intraoperative techniques. In practice, this distinction is less informative, as some lesions may be amenable to either preoperative mapping or intraoperative targeting, and multiple methods can be used in the same case. Additionally, no consensus exists on the superiority or inferiority of different localization techniques: the use of each technique, and each technique’s applicability to a specific case, is dependent on the practice patterns and institutional capabilities of each surgeon. Therefore, careful appraisal of the evidence in the context of the resources available is key. The final section is dedicated to advances in localization that are not yet widespread in practice.

## 2. Preoperative Techniques

Regardless of the planned or favored localization approach, every case starts with a preoperative assessment. Factors including nodule size, number, composition, and anatomic location inform every approach to planning a resection. Resecting a nodule not likely to be palpable often requires preoperative adjuncts to guide the operation. In cases requiring precise margin mapping or complex segmentectomy, or when a lesion is likely to be difficult to locate and >2 cm from the pleural surface, preoperative marking can be a valuable addition.

### 2.1. “Classical” Computed Tomography (CT)-Guided Preoperative Localization

The earliest approaches to lesion localization involve preoperative marking techniques. One example is CT-guided percutaneous needle localization, a technique which involves presurgical transthoracic placement of metallic wire directly into a target lesion. This provides visual cues with which the surgeon directly identifies the area within the lung parenchyma harboring the known lesion. Retrospective data reports safety and successful localization rates in the range of 97%. However, drawbacks including personnel requirements, cost, patient-specific restrictions, and the low but often intolerable rate of complications including pneumothorax, bleeding, and air embolism all exist [7]. Alternatively, thoracic surgeons may utilize the preoperative placement of identifiable alternatives such as dyes (methylene blue or ICG) that provide real-time intraoperative visualization during resection, radio-opaque substances (barium, lipiodol) assessed with intraoperative fluoroscopy, or radioisotopes (technitum-99) assessed with intraoperative radiotracer detection [8,9]. Similar to hookwire/needle localization, the risks of preoperative pneumothorax and adverse injection of critical structures persist; additionally, limiting factors in the use of localizing agents include diffusion of the agent from the area of localization, agent-induced inflammatory reaction, and the potential to distort the histology of the resected specimen [10]. In all, “classical” or conventional CT-guided localization can confer disadvantages but may still prove useful in select institutions.

### 2.2. Virtual-Assisted Lung Mapping (VAL-MAP)

VAL-MAP is a comprehensive preoperative protocol that uses preoperative imaging, bronchoscopic dye injection, and post-injection repeat CT with three-dimensional reconstruction, followed by short-interval surgery. Relative to other preoperative marking techniques, VAL-MAP has lower complication rates (4% pneumothorax versus 20–40%), can perform multiple markings and can identify subtle tomography of lung segments for extended or complex sublobar resections [11]. In earlier iterations of this technique, indigo carmine was the preferred agent but was plagued by difficulty in dye identification on post-bronchoscopic CT reconstruction and in the OR [12]. In the past decade, ICG-VAL-MAP has improved in its detection rates, which exceed 99% and are reported to result in over 97% when multiple marks are identified intraoperatively [13]. Novel prospective protocols coupling VAL-MAP with dye markings (VAL-MAP 2.0) and dual-agent VAL-MAP with indigo carmine plus ICG (VAL-MAP DS) have reported similarly positive success rates [14,15]. Although VAL-MAP has improved in its periprocedural complication rates and detection sensitivity, it remains a resource-intensive and institution-specific technology, and limited data has been published on its widespread adoption, applicability outside select institutions, or learning curve.

### 2.3. Electromagnetic Navigation Bronchoscopy (ENB), Robotic-Assisted Bronchoscopy

**Figure 1:**
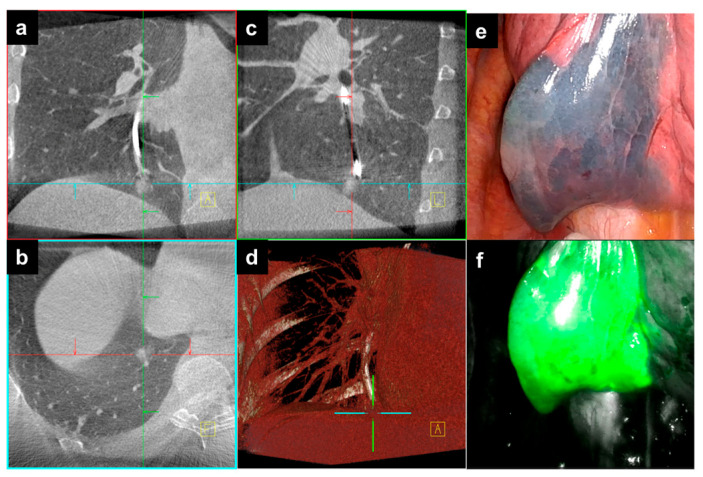
Intraprocedural cone-beam computed tomography (CBcT) utilized in concert with robotic bronchoscopy to localize a 15 mm right lower lobe ground-glass nodule and instill local methylene blue and indocyanine green. (**a**): coronal, (**b**) axial, (**c**) sagittal, and (**d**) real-time 3D reconstruction. Intraoperative appearance of (**e**) methylene blue and (**f**) indocyanine green.

Both traditional CT-guided localization techniques and VAL-MAP are limited in allowing access to deep or centrally located nodules and can fail to clearly define adequate resection margins in the absence of technology-assisted intraprocedural targeting. In contrast, ENB couples preoperative CT-assisted bronchoscopic marking with ICG, similar to VAL-MAP, with a navigational sensor allowing for more precise injection either at the site of the primary tumor or in a circumferential pattern defining adequate resection boundaries. In single-institution prospective studies, diagnostic yield across marking techniques was 97.5% with *N* = 80 patients [16]. However, in the large-scale multi-institutional NAVIGATE cohort with *N* = 537 patients with confirmed malignancy, diagnostic yield upon interval resection in lesions < 20 mm in diameter was only 62% [17], suggesting a significant heterogeneity in the institutional and technical factors resulting in successful localization. An alternative to EMB that leverages precision techniques and cone-beam CT (CBCT) to enhance targeting is shape-sensing robotic-assisted bronchoscopy (ssRAB). With ssRAB, a navigational display and catheter stabilization aim to improve inter-operator variability (Figure 1). In a single-institution retrospective study of over 200 patients, ssRAB outperformed ENB in diagnostic yield upon resection: 66% for ENB and 89% for ssRAB, resulting in an OR of 4.72 [95% CI 2.05–10.85] [18]. However, this result was discordant with the RELIANT trial, a single-institution, cluster-randomized trial assessing the diagnostic yield of bronchoscopic biopsy using either ssRAB or ENB. This trial found no difference in diagnostic yield (75.5% in the ENB group versus 77.8% in the ssRAB group) [19]. In all the literature reporting on ENB or RAB, operator variability (or lack thereof) must be accounted for: experienced operators may skew the true diagnostic yield rate positively, while novice operators may lead to lower rates. As differing approaches to achieve the same goal of precision bronchoscopy, ENB and RAB are applicable in similar clinical scenarios, but their use remains institution- and operator-dependent.

### 2.4. Novel Fiducial Markers

Whether the target lesion is marked by VAL-MAP, ENB, or ssRAB, these techniques primarily rely on the demarcation of the target by injection of an imaging agent, or by placement of fiducial markers that require secondary intraoperative imaging, either by fluoroscopy or intraoperative ultrasound. Especially in instances in which the operator marking the lesion preoperatively is different than the surgeon performing the resection, these techniques can lead to uncertainty, pose procedural hazard, and add logistical constraints. To better localize deep lesions and minimize logistical complexity, investigators from Kyoto University developed a radiofrequency identification (RFID) marking system as a novel fiduciary. Their RFID marker, inserted preoperatively under CBCT guidance, is localized with a probe that provides auditory cues to the surgeon. Purported advantages include a detection depth >2 cm, the threshold of detection for most localization dye [20]. In a retrospective comparison of their RFID probe to patients undergoing VAL-MAP with ICG dye, patients were more likely to undergo successful wedge resection without the need for a second wedge resection due to incomplete resection or positive margin (100% [95% CI 92.1–100] versus 94.6% [85.4–98.2%]) and less likely to require intraoperative palpation to confirm nodule localization (23% versus 77%) [21]. An additional approach to modernizing fiducial marker placement, without a transbronchial approach, combines the use of ICG with an embolization coil: preoperatively, ICG-soaked coils (ICG-Cs) are placed transthoracically. This combined approach allows for a longer time course of ICG marking, minimizing leaks to surrounding tissues, and is reported to maintain its viability as a localization technique as far out as 10 days post-procedure. Secondly, in the event that the ICG is too deep to localize, intraoperative fluoroscopy can serve as a backup option. However, in a multi-institution case series involving 19 patients, the asymptomatic pneumothorax rate was 19% [22]. These modalities require further evidence before widespread adoption. A practical decision-making algorithm to the localization of lung nodules can be found in Supplementary Figure S1.

## 3. Intraoperative Techniques

Though preoperative techniques can allow for enhanced presurgical planning, target discrete lesions, and improve surgical localization, these procedures can depend on the expertise of collaborative interventional staff, require increased resource utilization, result in complications, fail to localize the target lesion, and miss synchronous lesions. Intraoperative imaging techniques can allow for a consolidation of resources and mitigate the risk of perioperative complication. Methods like ultrasound, fluoroscopy, and intraoperative CBCT can help to localize a fiduciary placed preoperatively, while intraoperative molecular imaging can be helpful in identifying known or occult lesions within 2 cm of the pleural surface.

### 3.1. “Classical” Intraoperative Imaging

The approaches above including preoperative needle localization and preoperative injection of radio-opaque substances necessitate the use of intraoperative fluoroscopy. In addition, intraoperative ultrasound (IUS) is a tried and tested real-time, non-invasive, non-ionizing, and cost-effective adjunct. With the use of high-frequency ultrasound probes, solitary pulmonary nodules <2 mm can be identified in optimal conditions. Further, evidence exists to suggest that preoperatively identified ground-glass opacities (GGOs) can be identified upon intraoperative ultrasound with high sensitivity in an open approach (53/53, 100%), including 34/53 GGOs that were nonpalpable intraoperatively [20]. However, ultrasound technology is limited by the poor diffusion of sound waves across air spaces, necessitating complete intraoperative lung collapse and limiting its efficacy in emphysematous patients. In modern applications of robotic-assisted surgery, the necessity for robot-compliant ultrasound attachments can limit the previously seen cost advantages, but it does remain an option.

### 3.2. Intraoperative Cone-Beam CT (CBCT)

In contrast to the preoperative use of CT for wire or dye placement or as an adjunct to EMB, CBCT is an emerging modality in which low-dose, focused CT scans can be obtained intraoperatively (or intraprocedurally, if CBCT is used as an adjunct to bronchoscopic intervention) and real-time 3D reconstructions can be generated [21]. While the intraoperative use of CBCT has been historically limited given the technical and logistical constraints of equipment setup in an already crowded OR, the emergence of a smaller, “portable” CBCT with a C-arm similar to intraoperative fluoroscopy has allowed for early reports of intraoperative nodule localization at the subcentimeter level [22,23]. Intraoperative CBCT is a novel application of a technique already in use, but its specific use cases and logistical challenges require a clearer definition.

### 3.3. Fluorescence-Guided Surgery and Intraoperative Molecular Imaging (IMI)

**Figure 2:**
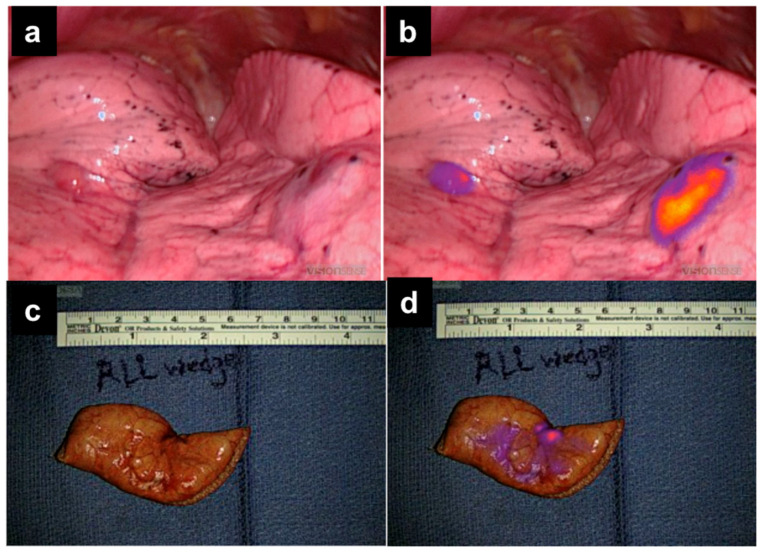
Intraoperative molecular imaging with the NIR contrast agent, second-window indocyanine green (ICG), TumorGlow. (**a**) White light view of surgical field. (**b**) NIR light demonstrating high fluorescence of an occult synchronous lesion (**left**) and primary lesion (**right**). (**c**) Ex-vivo right lower lobe wedge resection specimen under white light. (**d**) NIR light confirming fluorescent signal within the resected specimen, consistent with tumor localization. Images courtesy of Sunil Singhal, MD, Division of Thoracic Surgery, University of Pennsylvania.

Intraoperative molecular imaging (IMI) is an advanced surgical technique using specialized tracers—fluorescent or radioactive agents injected before surgery—to highlight cancer cells in real time. It enables surgeons to visualize tumor margins, identify lymph nodes, and differentiate healthy tissue from malignant ones, improving surgical precision and ensuring more complete tumor removal. One example is the near-infrared (NIR) contrast agent indocyanine green (ICG). ICG is an FDA-approved imaging agent originally designed to assess vascular perfusion imaging (at low doses with an interval of seconds to minutes) and biliary tract delineation (at higher doses with an interval of one to two hours). On a longer time course associated with the second-window technique (TumorGlow), ICG was found to accumulate in tumor tissue due to dysregulation in tumor-associated capillary networks allowing for enhanced permeability and retention (EPR) [24] (Figure 2).

In a prospective Phase I clinical trial published in 2019, our group demonstrated that non-primary pulmonary nodules, but not NSCLC, would fluoresce after low-dose ICG (1–3 mg/kg) 24 h before resection, and that after higher doses (4–5 mg/kg), both non-NSCLC cancers and NSCLC would fluoresce. These effects were relatively tumor-specific, with tumor-to-background (TBR) ratios at low doses of 3.19 for non-NSCLC cancers and 1.49 for NSCLC. At higher doses, TBR was not significantly different (3.21 vs. 2.70) [25]. Despite promising results in this trial, concurrent data published by other groups suggested a false-positive rate of approximately 12% (9/76 nodules) due to peritumoral or off-target increased parenchymal permeability due to benign or cancer-associated inflammation. Importantly, of the nine nodules that were not detectable by either white light or preoperative CT scan, 4/9 (44%) were true positive occult malignancies, while 5/9 (55%) were false positives. This effect also limited the ability of the surgeon to precisely delineate margins, as peritumoral inflammation would fluoresce [26]. Since 2020, these limitations and the emergence of an alternative agent have precluded ICG from widespread use; however, emerging research from Wang et al. suggests that intraoperative administration of inhaled ICG can yield almost double the median TBR (7.10, IQR 3.94–12.62) versus our and other intravenous administrations [27].

**Figure 3:**
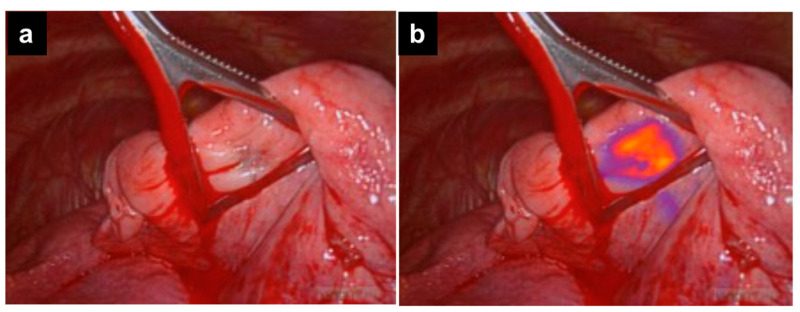
Nodule localization with (**a**) white light or (**b**) folate-receptor alpha-targeted IMI agent, pafolacianine.

In contrast to ICG, the development of targeted small molecules conjugated to fluorophores affords enhanced specificity for cancerous tissue. In the search for a candidate molecular target highly expressed in NSCLC and minimally expressed or absent in healthy lungs, folate receptor alpha (FRα) was identified as upregulation was present in 87% of pulmonary adenocarcinoma and in 13–57% of squamous cell carcinoma (SCC) [28,29]. As such, pafolacianine (previously called OTL38) was developed by OnTarget Laboratory (West Lafeyette, IN, USA). This agent involves a folate moiety covalently linked to an NIR fluorophore, (λex = 776 nm, λem = 793 nm). From 2015 to 2016, Phase I clinical trials evaluating pafolacianine in NSCLC demonstrated in situ fluorescence in 80% of patients (16/20). In total, 81% (17/21) of the nodules were localized with a mean TBR of 2.9 (IQR 2.1 to 4.2). When coupled with preoperative positron emission tomography (PET) in a multi-institutional prospective pilot study and with dose and timing optimization, pafolacianine improved its diagnostic yield and identified 56 of 59 (94.9%) malignant nodules identified by PET and nine additional subcentimeter malignant lesions not identified by PET [30,31]. In a Phase II trial of 92 eligible patients, pafolacianine identified 10 synchronous cancers undetected in preoperative workup, located 11 lesions that could not otherwise be visualized, and on the back table, revealed eight positive margins with histologic confirmation (Figure 3). Benefits were notably observed most frequently in patients with subcentimeter lesions or GGOs and in those patients undergoing complex sublobar resections [32]. Since these trials, the usage of pafolacianine has increased, and other groups have validated these findings: in a prospective study of the utility of pafolacianine in patients with GGOs, intraoperative molecular imaging detected 100% of malignant lesions, with a median lesion diameter of 14 mm, and changed surgical management (by upgrading to lobectomy) in 10% of cases [33].

In 2023, the ELUCIDATE trial, an open-label, multicenter Phase III randomized controlled trial, compared white light plus IMI with pafolacianine against white light only (standard-of-care) using a 10:1 treatment to control ratio. Patients enrolled were >18 years old with either suspected primary or metastatic lung cancer (*N* = 111). The primary efficacy endpoint was the proportion of IMI participants with a clinically significant event (CSE), defined as: (1) the removal of one or more primary lung nodules detected only by NIR examination, undetected by white light or palpation, and confirmed by histologic exam to be non-benign; (2) the removal of one or more synchronous lesions detected by only NIR examination and confirmed by histologic exam to be cancerous; or (3) the identification of a cancerous close margin of 10 mm or less under NIR, confirmed by histologic examination. Ultimately, 53% (53/100) of patients in the treatment arm achieved at least 1 CSE, with a total of 65 CSEs: 19% (19/100 patients) had localization not achieved by standard techniques, 8% (8/100 patients) had identification of synchronous lesions, and 38% (38/100 patients) had detection of fluorescence within 10 mm of the closest resection margin. Of the 53% of patients who had at least one CSE, 43 patients (81%) had one CSE, while 10 (19%) had two or more [34]. A post hoc analysis further characterized the 29 lesions in 23 patients identified exclusively with IMI: the majority were subcentimeter lesions (2 mm–20 mm, median 0.5 cm); 58% (17/29) were in the same lobe as the primary target nodule, whereas 42% (12/29) were found in an ipsilateral alternate lobe. However, only 34% (10/29) of the resected lesions were found to be cancerous on subsequent histopathologic review: seven synchronous primary adenocarcinomas, and three metastatic lesions, all from one patient with a history of chordoma; 26 of 29 lesions were managed with wedge resection [35]. Therefore, 19/29 (67%) of the additional resections that were performed led to benign diagnoses, and univariate analysis did not distinguish any predictors of the likelihood of malignancy. These data highlight a pitfall in the use of IMI: each surgeon needs to weigh the patient-specific risks and benefits as they consider the extension of their resection based on the IMI signal.

### 3.4. IMI in Metastasectomy

An additional application of IMI is the use of fluorescence in detecting and localizing occult pulmonary metastases in the context of metastasectomy. Resection of solitary metastatic lesions in lung or small-volume oligometastatic pulmonary disease is increasing in utilization: the most common primary tumors that necessitate metastasectomy are colorectal, lung, breast, renal, and melanoma, in that order, and survival in groups undergoing pulmonary metastasectomy is generally improved [36]. Often, the progression of disease in the lung after pulmonary metastasectomy can complicate cancer treatment: in the context of sarcoma, up to 35% of patients undergoing metastasectomy specifically for Ewing’s Sarcoma experience a pulmonary relapse necessitating reoperation or salvage treatment [37]. To assess whether IMI can improve the detection of occult metastases, our group conducted an open-label prospective clinical trial in which 52 patients with a history of sarcoma with prior disease remission and a primary nodule detected on surveillance underwent VATS or open thoracotomy with IMI guidance using second-window ICG. We found that in 31/52 patients (59%), IMI detected additional lesions, including some patients with multiple lesions. Median progression-free survival was significantly increased to approximately 8 months compared to historical controls, but survival was more likely to depend on disease burden at the time of surgery, and the design of the trial was not sufficient to generate strong oncologic conclusions [38]. Other groups have demonstrated the feasibility and efficacy of utilizing IMI for pulmonary metastasectomy in cancers like hepatocellular carcinoma, where Wang et al. showed a 90% rate of metastatic nodule fluorescence using ICG [39], and colorectal cancer, where Gutowski et al. demonstrated the feasibility of localizing lesions using a CEA-targeted fluorophore (SGM-101) [40].

### 3.5. Limitations of IMI with ICG and Pafolacianine

In contrast to forms of preoperative localization discussed in section two of this review, the use of optical contrast dyes including ICG and pafolacianine do not necessitate major deviations from a surgeon’s standard perioperative workflow. In our experience, pafolacianine can be administered in the preoperative holding area with minimal adverse events. Intraoperatively, most surgeons are already practiced in using an NIR camera, and no additional surgical steps are required. In the ELUCIDATE trial, IMI added a median of 2 min to the procedure and a maximum of 23 min [35]. However, both ICG and pafolacianine require specialized NIR-capable cameras to visualize fluorescence, and the excitation/emission wavelengths of these fluorophores are different. While ICG-capable cameras are generally available in most hospitals, cameras available to detect pafolacianine are less common.

False positives are a problem. Both ICG and pafolacianine generate false positives in areas of active inflammation, although for different hypothesized reasons. In ICG, enhanced capillary permeability induced by inflammation allows for the EPR effect to retain contrast. Conversely, in FRα-targeted agents, cross-reactivity between FRα, expressed at low levels in benign pulmonary epithelium and high levels of adenocarcinoma, and FRß, restricted to immunocytes, allows for nonspecific fluorescence in areas high in macrophage content like granulomas [41].

In contrast, ICG and pafolacianine exhibit false negatives for the same central reason: tumor size and tumor depth. Both have a minimum nodule size of approximately 5 mm, and a maximum depth from the pleural surface of approximately 2 cm. As a function of the properties of light, the mean fluorescent intensity of a sample decreases logarithmically with increases in depth past 1 cm [42]. Investigators from the University of Toronto are working to improve these limitations by coupling advances in ENB and RAB platforms to access central lesions: preclinical work in an ex vivo swine model has demonstrated a proof-of-concept that pafolacianine coupled with transbronchial tumor localization can visualize deep peribronchial tumors [43].

In a multivariate regression model surveying 309 patients undergoing resection with pafolacianine, factors were associated with a negative odds ratio of successful localization with IMI: a slight but significant decrease in odds with increased smoking pack years and distance from the pleural surface. Though significant, the absolute risk ratio was likely clinically insignificant when comparing patients at or below 40 PY (OR = 0.99, [95% CI 0.98–0.99]) and compounded as PY exceeded 40 and approached 120. As previously noted, distances from the pleural surface of >8 mm increased the OR of negative localization (OR 0.26, [95% CI 0.08–0.88]) [44].

At this point, the long-term impact of adjunctive IMI for pulmonary nodule localization, especially regarding its oncologic outcomes, lacks sufficient evidence. Earlier Phase I and Phase II trials using pafolacianine were non-randomized safety and feasibility studies conducted at experienced institutions developing the technology and were thus subject to bias; thus, these data should be viewed in context. The more recent ELUCIDATE trial, though randomized and conducted across 12 academic centers, had limited interval follow-up and was conducted in a 10:1 treatment-to-control ratio, and trial-specific follow-up was unlikely to be powered to detect oncologic difference. Critics may point to the 62% false-positive rate in IMI-alone detected lesions and contend that the risks of additional wedge resections and resultant loss of lung capacity outweigh the benefits of obtaining a complete oncologic resection in primary patients or minimal residual disease paradigm in patients with metastatic pulmonary burden.

## 4. Emerging Localization Approaches for NSCLC

### 4.1. Cathepsin-Targeted Agents (VGT-309)

Cathepsins are proteolytic enzymes upregulated in cancer cells and the external tumor microenvironment, where they promote cell invasion and the establishment of an oncogenic niche. VGT-309 is a small-molecule homolog of cysteine with an indocyanine green domain that acts as a substrate to an activated cysteine cathepsin. When the cathepsin cleaves the molecule, it releases the fluorescence-quenching moiety from the indocyanine green and allows for NIR fluorescence. Two distinct advantages of VGT-309 over existing optical contrast agents exist: first, VGT-309 fluoresces in the ICG channel, which is much more common in hospitals across the country than cameras capable of the excitation and detection of pafolacianine. Second, unlike FRα and pafolacianine, in which a positive TBR can be interpreted as a binary determination [41], as high FRα positivity is not necessarily correlated with more aggressive or locally advancing disease, VGT-309 has been shown to correlate fluorescent intensity with metabolic and oncologic activity, allowing for the assessment of local spread or lymph node metastasis [45,46]. In a recently completed phase 2 clinical trial, our group demonstrated safety and efficacy: of 40 patients undergoing surgical resection, 42.5% (95% CI 27.0–59.1%) had at least one CSE. In this cohort, VGT-309 outperformed the post hoc analysis of the lesions detected with IMI in the ELUCIDATE trial with a 77.3% true positive rate for cancerous tissue (34/44 lesions detected, relative to 38% or 11/29 in ELUCIDATE) [34]. Further trials utilizing VGT-309 are ongoing, including VISUALIZE, (NCT06145048).

### 4.2. pH-Activated Agents (Pegsitacianine, ONM-100)

Evidence exists that the increased anaerobic respiration and dysregulated metabolism induced by the Warburg effect common to nearly all cancers lead to the expulsion of protons and an acidic tumor microenvironment. In this context, our group developed an optical agent that was inert at physiologic pH and activated at an acidic pH, thus fluorescing only at pH levels supraphysiologic for lung parenchyma. Despite promise in preclinical models, in a Phase II multicenter clinical trial using this agent, pegsitacianine performed less well, as it did not consistently label known lesions (31.6%, 6/19 lesions) and was less tolerated, with four infusion reactions that resolved upon discontinuation [47].

### 4.3. Antibody Conjugates

Investigations led by Dr. Lui at Stanford University and Dr. Bharadwaj at the University of Saskatchewan are recruiting patients for Phase I/II studies investigating the safety and efficacy of an investigational dye conjugate, IRDye800CW, with either panitumumab or nimotuzumab, both humanized monoclonal antibodies targeting the epidermal growth factor receptor (EGFR), and expect to mature results in the coming years (NCT03582124, NCT04459065). The development and use of these agents would likely target select cancers with high specificity but have limited applicability in antigen-negative or unsequenced cancers: EGFR is a highly studied driver mutation with higher rates of positivity in adenocarcinomas, people of East Asian origin, never-smokers, and females, but an aggregate rate of approximately 15% of adenocarcinomas in North American patients [48].

### 4.4. Future Directions: IMI in Preclinical and Ex Vivo Models

Investigators worldwide are working in the IMI space to improve protocols and introduce novel solutions: each of the following projects was published in the past year. One such effort by Chao et al. employs a nanoparticle containing an iron oxide core coupled to cetuximab, an EGFR-targeted monoclonal antibody, and an NIR tracer (SPIOs-EGFR-IRDye800), to show in murine and ex vivo models the dual utility of the agent as a form of targeted MRI contrast dye and intraoperative IMI agent that can be used after a single dose for both preoperative imaging and intraoperative localization [49]. Another construct utilizes fluorescent dyes with a biosimilar motif to fibroblast-activating protein (FAP), a well-studied transmembrane protein with a strong expression in cancer-associated fibroblasts characterized to promote tumorigenesis. This construct is especially promising given advancements made with preoperative FAP-PET, again providing a dual-modality agent for preoperative and intraoperative planning [50]. Lastly, our group defined the sodium–glucose cotransporter 2 (SGLT2) as a molecular marker specific to early-stage lung adenocarcinoma and modified the already-FDA-approved backbone of the antidiabetic SGLT2 inhibitors to incorporate an optical agent comparable to ICG and demonstrated a mean TBR of 2.23 in murine models [51].

## 5. Limitations

### 5.1. Limitations of This Review

This descriptive review is based on our experience, knowledge, and literature search on localization techniques in lung cancer with emphasis on studies published in the past 10 years. Because our process was not systematic, it risks selection bias. The data presented is often derived from small prospective trials of select patients at centers that have more experience and more technical capability than the average hospital. In all techniques, inter-operator variability limits conclusive statements about generalizability.

### 5.2. Limitations of Localization Techniques

Throughout the techniques covered, none is clearly superior: each has potential for benefit and trade-offs at clinical and system levels. Each localization technique should be weighed carefully in the context of the case, cost, resources available, logistical constraints, collaborating physicians and staff, and background and training of the surgeon. For some, standardization of a specific workflow may reliably improve localization and streamline the operation; for others, localization may only be worthwhile in specific or challenging cases. Ultimately, the judgment of when or if using a technique will be beneficial is dependent upon the surgeon and the institution. A consolidated summary of the advantages and limitations for each localization technique available today is presented in Table 1.

## 6. Conclusions and Perspective

The landscape of the surgical treatment of lung cancer is trending towards a greater proportion of limited resections of smaller lesions. In this context, strategies to localize a lesion with precision will allow for a more thorough calculation of the risks and benefits of the extent of resection in each case. During a preoperative assessment, if the lesion is unlikely to be palpable or obvious with white light visualization, adjunctive localization techniques should be considered. Depending on the institutional capabilities and training of the surgeon, one or more of the techniques described can be employed: navigational bronchoscopy techniques like VAL-MAP, ENB, or RAB can mark even mid-peripheral lesions with high degrees of accuracy. Fiduciaries and intraoperative adjuncts like ultrasound or fluoroscopy may suffice. Intraoperative molecular imaging can streamline the care of the patient and the workflow of the surgeon, and may confer benefits in metastatic or multifocal disease, although trade-offs and limitations should be carefully considered.

## Figures and Tables

**Table 1:** Summary of available localization techniques.

Technique	Use Case	Advantages	Disadvantages
CT-Guided Localization	Localization of lesion that is accessible by transthoracic injection	- High localization success rates - Direct visual guidance - Most commonly available	- Risk of pneumothorax, bleeding, and air embolism - Wire dislodgement - Coordination of perioperative care - May require intraoperative fluoroscopy
VAL-MAP (Virtual-Assisted Lung Mapping)	Complex sublobar resections and localization of subtle lesions	- Sophisticated anatomical delineation - High detection sensitivity	- Resource-intensive - Institution-specific expertise required
Electromagnetic Navigation Bronchoscopy (ENB)	Precision localization and biopsy of pulmonary nodules	- Navigational guidance allows for subsegmental accuracy	- Variable diagnostic yield - Operator-dependent performance - Institution-specific expertise required
Robotic-Assisted Bronchoscopy (RAB)	Precision localization and biopsy of pulmonary nodules	- Enhanced catheter stability and targeting precision	- Requires specialized equipment and expertise - Cost
Novel Fiducial Markers (RFID, ICG-Coils)	Localization of deep lesions	- Auditory guidance, to localize deep lesions - Prolonged localization capability	- Not yet widespread, may be unavailable - Risk of pneumothorax with transthoracic placement
Intraoperative Ultrasound	Real-time intraoperative localization of nonpalpable nodules and GGOs or pre-placed fiduciaries	- Non-ionizing - Cost-effective - Highly sensitive in optimal conditions	- Limited by emphysematous lung or incomplete lung collapse
Intraoperative Cone-Beam CT (CBCT)	Real-time intraoperative imaging and localization	- 3D reconstruction and subcentimeter localization capability	- Logistical and equipment constraints - Cost
Indocyanine Green (ICG) IMI	Fluorescence-guided localization of tumors and metastases	- Real-time visualization - Widely available imaging systems	- Limited depth penetration, nodule size < 5 mm - False positives from inflammation
Pafolacianine IMI	Targeted fluorescence imaging of NSCLC and occult lesions	- Real-time visualization - Sensitivity for occult lesions - Margin assessment capability	- Requires specialized NIR cameras - Limited depth penetration, nodule size < 5 mm - False positives from inflammation - Low specificity for occult lesions

## Data Availability

All data referenced can be found in the cited publications.

## Supplementary Materials

The following supporting information can be downloaded at: https://www.mdpi.com/article/10.3390/cancers18121915/s1, Figure S1: Perioperative localization of pulmonary nodules clinical decision-making algorithm.
