# Supplementary figures and images for: Intraoperative Nodule Localization in Non-Small-Cell Lung Cancer: Existing and Emerging Techniques
Source: Cancers (Basel). 2026 Jun 12;18(12):1915. doi: 10.3390/cancers18121915 (PMC13296510; doi:10.3390/cancers18121915)

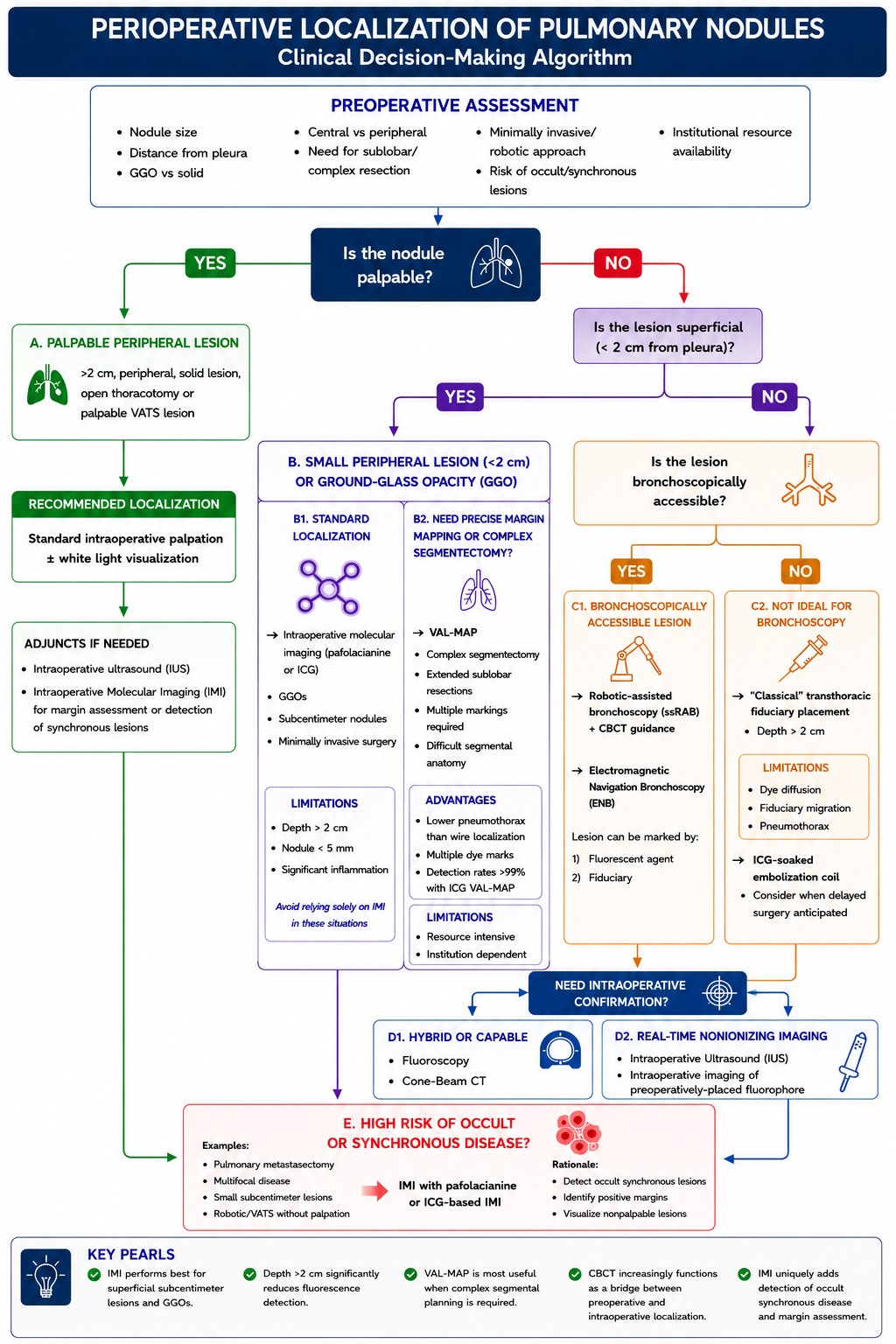

Supplement: Supplementary file 1 [file cancers-18-01915-s001.zip › cancers-4296679-supplementary.jpg]
